# HLA-G Is Widely Expressed by Mast Cells in Regions of Organ Fibrosis in the Liver, Lung and Kidney

**DOI:** 10.3390/ijms222212490

**Published:** 2021-11-19

**Authors:** Nicolas Mouchet, Nicolas Vu, Bruno Turlin, Nathalie Rioux-Leclercq, Stéphane Jouneau, Michel Samson, Laurence Amiot

**Affiliations:** 1 Univ Rennes, INSERM, CNRS, Biosit, Core Facility H2P2, F-35000 Rennes, France; nicolas.mouchet@univ-rennes1.fr; 2 France BioImaging, Biogenouest, IBISA, F-35000 Rennes, France; 3 Univ Rennes, INSERM, EHESP, IRSET (Institut de Recherche en Santé, Environnement et Travail)-UMR_S 1085, F-35000 Rennes, France; nicolas.vu@univ-rennes1.fr (N.V.); michel.samson@univ-rennes1.fr (M.S.); 4 Univ Rennes, CHU Rennes, F-35000 Rennes, France; bruno.turlin@chu-rennes.fr; 5 Univ Rennes, CHU Rennes, INSERM, EHESP, IRSET (Institut de Recherche en Santé, Environnement et Travail)-UMR_S 1085, F-35000 Rennes, France; nathalie.rioux-leclercq@chu-rennes.fr (N.R.-L.); stephane.jouneau@chu-rennes.fr (S.J.)

**Keywords:** fibrosis, HLA-G, mast cells, follicles, B lymphocytes, antifibrotic

## Abstract

We previously demonstrated that mast cells expressing HLA-G are associated with regions of hepatitis C virus-induced liver fibrosis. Here, we aimed to determine whether HLA-G expression in mast cells is specific to viral etiology, the liver, or to the general process of fibrosis. We enumerated HLA-G^+^ cells and mast cells by the immunohistochemistry of (i) liver blocks from 41 cases of alcoholic cirrhosis, (ii) 10 of idiopathic pulmonary fibrosis (IPF), and (iii) 10 of renal fibrosis. The nature of the HLA-G^+^ cells was specified by multiplex immunofluorescence using software. More than half of all HLA-G^+^ cells were mast cells in fibrotic areas of alcoholic cirrhosis and IPF. In the kidneys, subjected to fibrosis, the HLA-G^+^ cells were indeed mast cells but could not be counted. Moreover, in certain cases of the liver and lung, we observed a number of cellular nodes, which were secondary or tertiary follicles, in which HLA-G was highly expressed by B lymphocytes. In conclusion, HLA-G^+^ mast cells could be observed in the fibrotic regions of all organs studied. Previous studies suggest a protective role for HLA-G^+^ mast cells against inflammation and fibrosis. The observed follicles with B lymphocytes that express HLA-G may also reinforce their antifibrotic role.

## 1. Introduction

Chronic diseases that lead to organ fibrosis are associated with significant mortality and morbidity, accounting for up to 45% of deaths in developed countries [1]. The prevalence of fibrotic diseases is steadily increasing and is an important public health problem.

Fibrotic diseases can affect all organs, such as the liver, kidneys, lungs, and heart. Pathological fibrosis is characterized by the exaggerated deposition of components of the extracellular matrix (ECM), such as collagen. Such ECM accumulation destroys the normal architecture of the organ and leads to organ dysfunction and failure with alteration of the specialized functions of each organ, i.e., for the liver, the function of detoxification, for the lung, the function of gas exchange, and for the kidney, the function of filtration. In addition, fibrosis can promote the development of cancer. Transplantation to replace the fibrotic organ often represents the best therapeutic option.

The process of fibrosis occurs in response to an injury or tissue damage following a persistent or overly strong inflammatory response. Fibrosis, which is initially reversible, can evolve to an irreversible state [2]. The liver can be injured by viruses (hepatitis B, C, D, E), fungal toxins, parasites, auto-antibodies, a high-fat diet, and excessive alcohol consumption, which is a frequent etiology of liver fibrosis [3] and even the predominant cause in certain countries. When the insults are repeated, the liver develops chronic hepatitis with fibrosis followed by advanced fibrosis or cirrhosis, which is a predisposing state for hepatocellular carcinoma (HCC).

In the lung, idiopathic pulmonary fibrosis (IPF) represents the most common of a group of diseases that includes hypersensitivity fibrosis and rheumatoid lung. This chronic progressive fibrosing interstitial lung disease of unknown origin is rare, affecting three million people worldwide. However, IPF is associated with early death. Indeed, IPF leads to advanced respiratory failure and also represents an independent risk factor for lung cancer [4]. Lung transplantation should be considered as an option for young patients with advanced disease.

Renal fibrosis is the final common pathway of numerous progressive kidney diseases. The incidence of chronic kidney disease, leading to end-stage renal disease, has significantly increased, affecting 10% of the worldwide population, with high mortality [5]. In addition, patients with chronic kidney disease have an increased risk of developing kidney cancer (up to 10 times that of the general population), with frequent bilateral and/or multifocal damage [6].

Regardless of the organ that develops fibrosis, it is important to understand the mechanisms involved in its emergence to develop therapies to prevent it. In particular, chronic inflammation leads to liver or renal fibrosis, and controlling it may make it possible to limit its progression and the onset of organ failure or cancer. Cytokines and chemokines play a central role in both the orientation of the immune response and the maintenance of inflammation [7,8]. In addition to these immune molecules, other proteins, such as HLA-G, a class Ib HLA molecule well known for its immunomodulating properties, have been investigated [9]. We previously demonstrated that HLA-G is expressed by mast cells that are associated with the area of hepatitis C virus-induced liver fibrosis [10,11]. In the present study, we investigated whether HLA-G expression in mast cells is specific to viral etiology, the liver, or the process of fibrosis, irrespective of the organ. We characterized HLA-G expression in mast cells and immune cells on paraffin blocks of cohorts of 41 patients with alcohol-induced cirrhosis, 10 with IPF, and 10 with renal fibrosis. Precise identification of HLA-G-expressing cells was performed using quadruple immunofluorescence on paraffin sections and software that separately analyzes the fluorescence and merges it on three cases of liver alcohol-induced fibrosis and two of IPF.

## 2. Results

### 2.1. Quantitative Expression and the Nature of HLA-G^+^ Cells in Alcohol-Induced Fibrosis

An initial immunohistochemistry study was performed on a series of alcohol-induced fibrosis samples (n = 41), allowing the enumeration of HLA-G^+^ and CD117^+^ cells over the entire slides by semi-automatic enumeration using HALO software.

The results are summarized in Table 1.

HLA-G^+^ and CD117^+^ cells were counted over the entire surface of serial slides of 41 cases of alcoholic cirrhosis using HALO software with the appropriate algorithm following immunohistochemistry with 4H84, which recognizes HLA-G, and anti-CD117/c-kit. The mean, standard deviation, and range are indicated for the HLA-G^+^ and CD117^+^ cells: 555 ± 699 HLA-G^+^/(41–2686) and 200 ± 271 CD 117^+^ cells (12–1113).

**Figure 1:**
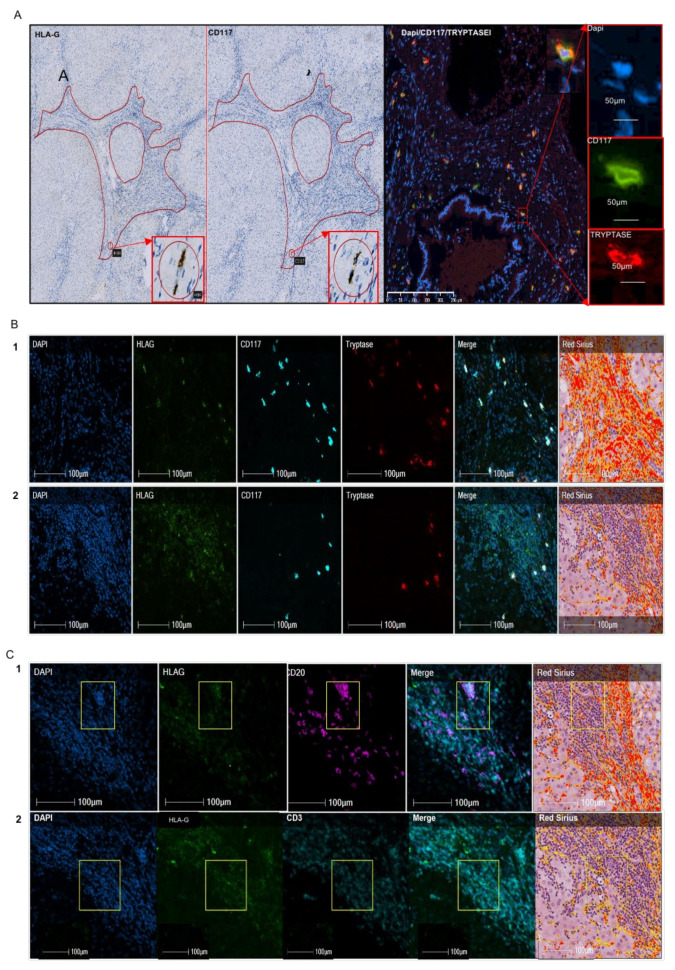
Nature of HLA-G cells on a representative case of liver fibrosis. (**A**) In the first paragraph, the two photos on the left show HLA-G and CD117 expression on a representative case of liver fibrosis out of 41 cases using immunohistochemistry, with 4H84 recognizing HLA-G and CD117 antibody recognizing c-kit cells. The slides were counterstained by Mayer hematoxylin. Fibrosis spans are surrounded by a red line. The areas in circles are topographically corresponding to HLA-G and CD117 stained slides. The rectangle shows a strong magnification of this areas indicating that the two HLA-G cells match the two CD117 cells. Moreover, they have a morphology compatible with a mast cell. The photo in double fluorescence (CD117/Mast cell tryptase) with the three photos on the right corresponding to the simple fluorescence (DAPI coloring the nuclei in blue, CD117 in green, mast cell tryptase in red) show a majority of cells co-labeled in yellow. The CD117 cells are largely mast cells. (**B**) Quadruple immunofluorescence on a representative case of liver fibrosis (out of three) using DAPI with nuclei blue, HLA-G (with 4H84 antibody) with green pseudo-color, CD117 in cyan pseudo-color, mast cell tryptase in red pseudo-color, and the composite image or merge. On the right, Sirius Red staining is represented. 1 and 2 are corresponding to two different areas of the liver, respectively fibrosis span and cell node. The majority of HLA-G cells co-express CD117 and mast cell tryptase in fibrosis (1). In contrast, the majority of HLA-G cells are not co-expressing mast cells, except for certain HLA-G cells in the periphery of the cell node (2), near or in fibrosis. (**C**) Quadruple immunofluorescence on the node cells of liver fibrosis shown in B2, with DAPI, HLA-G with green pseudo-color, CD20 with fuchsia pink, or CD3 with cyan pseudo-color, respectively, in 1 and 2. The Sirius Red coloration matching is on the right. The square shows a limited topographically area containing HLA-G positive cells and the corresponding cells in that zone. The composite image shows the pink co-labeling of HLA-G and CD20. No co-staining is observed between HLA-G with green pseudo-color and CD3 with cyan pseudo-color.

The morphological features of HLA-G^+^ cells in alcohol-induced cirrhosis topographically correspond to mast cells (Figure 1A). In certain cases, double labeling for CD117 and human mast cells was performed and showed that the hepatic CD117^+^ cells are mast cells in the fibrotic area (Figure 1A).

Three representative cases of alcohol-induced cirrhosis were studied by quadruple immunofluorescence and Sirius Red staining using HALO software to precisely identify the HLA-G^+^ cells. The average area of fibrosis corresponded to 19.4% of the liver tissue.

The identification of HLA-G cells^+^ showed 51% to be mast cells (Table 2; Figure 1A,1B1) defined by the human mast-cell tryptase + CD117− phenotype or human mast-cell tryptase + co-staining for CD117. Mast cells expressing HLA-G were located in the fibrotic areas (Figure 1B1). Conversely, 49% of the HLA-G^+^ cells were not mast cells nor CD117^+^ cells (Table 3) and generally appeared to be grouped together as nodes (Figure 1B2). Thus, other labeling was carried out on the different zones to identify these cells (Table 4). The repartition of the different types of HLA-G^+^ cells differed depending on the area of liver tissue examined (Table 3 and Table 4). Overall, 63% of the HLA-G^+^ cells in the fibrotic area appeared to be mast cells versus only 3% in the nodes. In addition, 68% of HLA-G^+^ cells in the nodes co-expressed CD20, a marker of B lymphocytes (Figure 1C1), and 3% CD3 (Figure 1C2).

HLA-G^+^ CD117^−^ mast cell tryptase HLA-G^+^ CD117^−^ HLA-G^+^ CD117^−^ mast-cell tryptase^+^, and HLA-G^+^ CD117^+^ mast-cell tryptase^+^ cells were counted in three representative cases of liver fibrosis using HALO software with the appropriate algorithm.

The cells were counted in two different areas of the alcoholic cirrhosis sample, i.e., fibrotic area and cell node, in a representative case of liver fibrosis using HALO software with the appropriate algorithm. No HLA-G^+^ cells co-express CD31, a marker of endothelial cells or CD1a, a marker of dendritic cells.

The absolute number of HLA-G^+^, CD3^+^ (T lymphocytes), CD20^+^ (B lymphocytes), and mast-cell tryptase^+^ (mast cells) cells was determined using HALO software with the appropriate algorithm on one representative case and is expressed per mm^2^.

### 2.2. Analysis of HLA-G^+^ Cells in IPF

**Figure 2:**
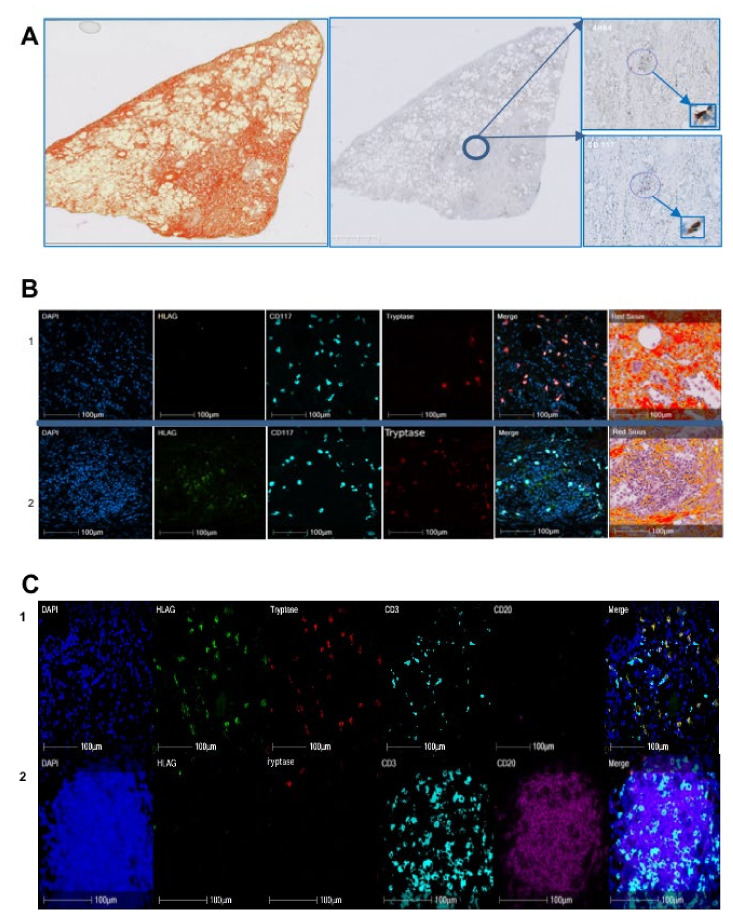
Nature of HLA-G cells on a representative case of IPF. (**A**) In the first paragraph, the photo on the left shows the Sirius Red coloration of one representative case out of ten IPF, the middle photo shows the counterstain by Mayer hematoxylin using immunohistochemistry. The areas in circles are topographically corresponding on HLA-G and CD117 stained slides with 4H84 recognizing HLA-G and CD117 antibody recognizing c-kit cells. The rectangles show a strong magnification of these areas, indicating that HLA-G cells do not match CD117 cells. (**B**) Quadruple immunofluorescence on a representative case of liver fibrosis out of two using DAPI with nuclei blue, HLA-G (with 4H84 antibody) with green pseudo-color, CD117 in cyan pseudo-color, mast cell tryptase in red pseudo-color, and the composite image or merge. On the right Sirius Red is represented; 1 and 2 are corresponding to two different areas of the lung, respectively the fibrosis span and cell node. (**C**) Quintuple immunofluorescence with DAPI, HLA-G with green pseudo-color, tryptase with red pseudo-color, CD3 with cyan pseudo-color, CD20 with fuchsia pink pseudo-color, on the same case of IPF shown in (**B**). (**C1**) is corresponding to the (**B1**) area. In (**C1**), the majority of HLA-G cells is corresponding to mast cells in the fibrosis area, whereas very rare mast cells are HLA-G in the node shown in (**C2**). The majority of the cells of the node are CD20. HALO analysis showed most of the HLA-G+ cells to be B lymphocytes.

In the first study performed on a series of IPF (n = 10) by immunohistochemistry, as previously described, we estimated the number of HLA-G^+^ cells to be 135 ± 62/mm^2^ and that of CD117^+^ cells to be 227 ± 134/mm^2^ (Table 5). The HLA-G^+^ cells did not match the CD117^+^ cells in lung fibrosis (Figure 2A). HALO analysis of quadruple immunofluorescence and Sirius Red staining showed the nature of HLA-G cells to be different depending on the histological area of the lung (Table 6). Indeed, 63% of HLA-G^+^ cells in fibrotic areas were mast cells (B1, C1), whereas they only comprised 7% of HLA-G^+^ cells in the nodes (B2, C2). Most of the HLA-G^+^ cells in the nodes co-expressed CD20 (Figure 2B).

HLA-G^+^ and CD117^+^ cells were counted for the 10 cases using HALO software with the appropriate algorithm, following immunohistochemistry. The mean, standard deviation, and range are indicated.

### 2.3. Analysis of HLA-G^+^ Cells in Renal Fibrosis

**Figure 3:**
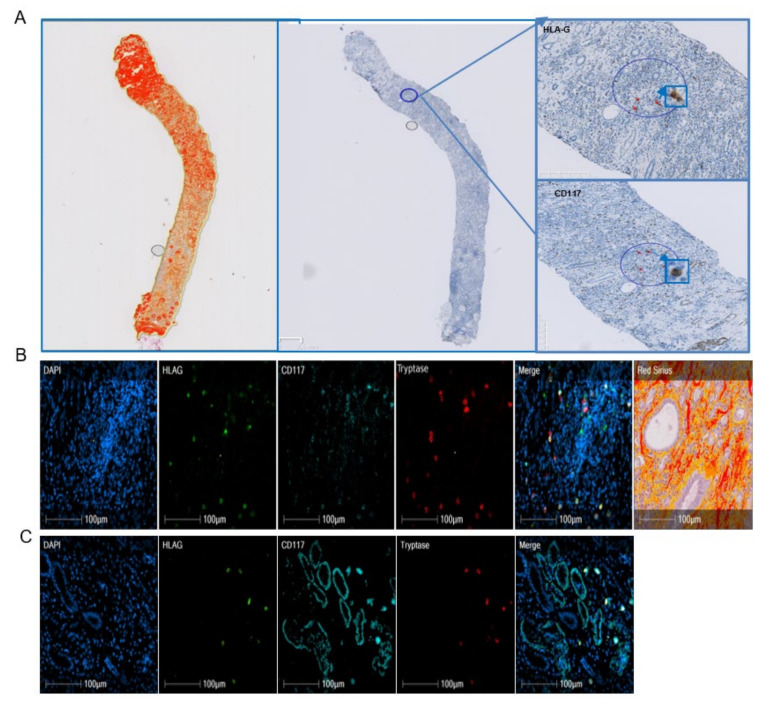
Nature of HLA-G cells on a representative case of renal fibrosis. (**A**) In the first paragraph, the photo on the left shows the Sirius Red coloration, the middle photo shows the counterstain by Mayer hematoxylin using immunohistochemistry. The areas in circles are topographically corresponding to HLA-G and CD117 stained slides, with 4H84 recognizing HLA-G and CD117 recognizing c-kit cells. Red arrows are positive cells. The rectangles show a strong magnification of these areas indicating that HLA-G cells do not match CD117 cells. (**B**) Quadruple immunofluorescence on a representative case of liver fibrosis using DAPI with nuclei blue, HLA-G (with 4H84 antibody) with green pseudo-color, CD117 in cyan pseudo-color, mast cell tryptase in red pseudo-color, and the composite image or merge. On the right, Sirius Red is represented. Cells stained yellow on the merge are corresponding to HLA-G+ mast cells. (**C**) The areas stained cyan are corresponding to numerous tubules only expressing CD117. Cells stained yellow on the merge are corresponding to HLA-G+ mast cells.

In the study of a series of 10 cases of renal fibrosis by immunochemistry, we found 133 ± 102 HLA-G^+^ cells/mm^2^ and 426 ± 207 CD117^+^ cells/mm^2^ (Table 7). There was no match between the HLA-G^+^ and CD117^+^ cells (Figure 3A).

HLA-G^+^ and CD117^+^ cells were counted for 10 cases of renal fibrosis using HALO software with the appropriate algorithm, following immunohistochemistry. The mean, standard deviation, and range are indicated for HLA-G^+^ and CD117^+^ cells.

Quadruple labeling with HALO software coupled to Sirius Red and morphology analysis showed substantial heterogeneity of the labeling on a section of kidney according to not only the anatomical region but also within the same anatomical compartment (Figure 3B,C). Thus, the enumeration and calculation of the percentage of HLA-G^+^ cells were not relevant in this organ. Indeed, microscopic examination showed strong staining of the circumference of tubules with CD117 that did not co-label with that of mast cells (Figure 3C). The HLA-G^+^ cells are mast cells and are located in the inflammatory interstitium (Figure 3B,C).

## 3. Discussion

The expression of HLA-G proteins was first demonstrated in cytotrophoblasts at the fetal–maternal interface [12]. Under basal conditions, its expression is largely restricted to specific tissues, such as the cornea [13], thymus [14], and β islets of the pancreas [15]. However, certain types of cells are also able to express it, such as bronchial epithelial cells [16], mesenchymal cells [17], cells of monocytic lineage [18,19,20], and erythroid and endothelial precursors [21], in peculiar conditions.

We previously demonstrated that mast cells can express HLA-G in the basal state, with increased expression in certain cytokine-rich environments, in particular, fibrotic liver tissue. We investigated whether HLA-G can be expressed by mast cells associated with liver fibrosis from another etiology or fibrosis in another organ by studying 41 cases of alcohol-induced liver cirrhosis, 10 of IPF, and 10 of renal fibrosis.

Infections, toxic and metabolic injuries, and idiopathic inflammatory diseases can promote the development of fibrosis because chronic injury induces an apoptosis of parenchymal cells which release profibrogenic and inflammatory cytokines such as TGF-β. The collagen-producing cells differentiate from the resident mesenchymal cells in response to the injury. Epithelial to mesenchymal transition is a phenomenon of cell transdifferentiation that is observed for cholangiocytes in liver, pneumocytes in lung, and tubular epithelial cells in kidney [22]. Apoptotic cells induce an increase in the concentration of TGF-β in all organs. However, specific features of fibrogenesis may be distinguished in the different organs. In the liver, apoptosis concerns hepatocytes, whereas it affects epithelial cells in lung and kidney [22]. Thus, resident fibroblasts in kidney and lung activate into a myofibroblast expressing a-SMA, collagen1, whereas liver myofibroblast retain their neural-specific markers [23].

In the liver, the hepatic stellate cells contribute more than 80% of all collagen-producing cells. In lungs, the damage of pneumocytes is associated to the apoptosis of endothelial cells. The role of inflammation in IPF is controversial. Typical IPF does not show an influx of inflammatory cells, but some authors suggest a role of inflammation in the differentiation of pulmonary fibroblasts into ECM-producing myofibroblasts [24]. Repeated alveolar epithelial lesions of unknown etiology and alveolar epithelial apoptosis are involved in IPF [25]. In the kidney and liver, myelomonocytic cells are recruited from the bone marrow and represent respectively 14 to 15% and 8 to 12% of the myofibroblasts. No reversibility of fibrosis is observed in the lung in contrast to the liver and kidney in the absence of injury and if the point of no return has not been reached. Indeed, inflammatory processes are limited in IPF, in particular at the early phase of the disease, whereas repeated alveolar epithelial lesions of unknown etiology and alveolar epithelial apoptosis can promote the proliferation and activation of pulmonary fibroblasts or myofibroblasts [25].

As for liver fibrosis, a failed wound-healing process of the kidney tissue after chronic, sustained injury leads to the production and secretion of proinflammatory cytokines, as well as TGF-β, which plays a key role in the fibrotic process. For example, in liver fibrosis, TGF-β, which is expressed as a minute amount in quiescent HSC, is quickly produced by this type of cells after liver injury. In addition to the HSC, other sources of TGF-β have been described as platelets, macrophages, hepatocytes, and also mast cells [26]. TGF-β1 is stored in the matrix in its latent form, and once activated, it promotes the transition from fibroblast to myofibroblast, which is fundamental for the fibrosis process. In addition, it inhibits ECM degradation by suppressing metalloproteases and promoting a natural inhibitor TIMP. Thus, it induces the production of ECM through SMAD3-dependent or non-SMAD-associated mechanisms [27]. Indeed, a mutual interaction exists between mast cells and TGF-β. TGF-β is a potent attractant for mast cells; indeed, the pathologic processes mediated by TGF-β are often associated with mast cell accumulation [28]. In addition, mast cells are one of the primary sources of IL-17 that drive TGF-β-dependent fibrosis [29]. TGF-β has been also reported to promote or suppress mast cells functions. Indeed, TGF-β inhibits the expression of the high-affinity IgE receptor Fc1RI, which activates mast cells [30]. On the other hand, it inhibits mast cell proliferation, degranulation, and the production of several effector molecules such as histamine and TNF-β [31]. Given the increase in MC in fibrosis, the effect of TGF-β on MC functions can be important in the regulation of inflammatory responses that maintain the fibrosis process.

As in hepatitis C virus-induced liver fibrosis, we found half of the HLA-G^+^ cells in alcohol-induced cirrhosis to be mast cells (Table 2) and only 34% of mast cells expressed HLA-G, with high individual variability shown by the standard deviation (Table 1). In addition, we observed a distinct repartition according to the region of the liver, in which 63–92% of the HLA-G^+^ cells in fibrotic regions were mast cells, whereas only 3–23% were mast cells in cellular nodes (Table 3). Similarly, a different pattern is observed for mast cells, since 92% of the mast cells in fibrotic regions expressed HLA-G, whereas only 23% expressed HLA-G in cellular nodes (data not shown). Thus, the expression of HLA-G is not restricted to the viral etiology of liver cirrhosis. Indeed, we obtained a similar result for lung. In IPF, 63% of HLA-G^+^ cells in fibrotic regions were mast cells, whereas only 7% of those in nodes were mast cells (Table 6). The cases of renal fibrosis were particular. As a result of the large number of tubules, it was not possible to properly count the cells in the fibrotic regions because mast cells had infiltrated the tubules. Only qualitative microscopic observation could be performed, showing a number of HLA-G^+^ cells to be mast cells, without being able to differentiate tubules from fibrotic regions (Figure 3). Thus, HLA-G appears to be expressed by mast cells in fibrotic disease through cell surface and intra-cytoplasmic molecules, irrespective of the organ. We have previously demonstrated that human mast cells in culture were able to produce soluble HLA-G forms in the conditioned medium at basal state and that secretion increased after stimulation with cytokines, including IL-10 [10].

The higher percentage of mast cells (more than half) expressing HLA-G in the liver and lung may be explained by the inflammatory components of fibrosis. Indeed, as innate immune cells, the number of mast cells increases in inflammatory conditions, and they can also release proinflammatory mediators [32].

The number of mast cells is elevated in fibrotic diseases. Indeed, mast cell density is higher in the lungs of patients with IPF than those with other lung pathologies [33] and normal lung. Similarly, human renal diseases are accompanied by an increase in the number of mast cells in the renal cortex, especially in the region of fibrosis [34], as mast cells are rarely observed in healthy kidneys.

Previous publications stated that mast cells are absent from or only sparsely found in normal human liver, lungs, and kidneys [35]. The progress of the knowledge on mast cells has shown that mast cells as innate immune cells can be observed in all the tissues, but they are more abundant at sites exposed to the environment. Moreover, they display a large repertoire of receptors allowing them to respond to stimuli and to interact with other cells [36]. Renal mast cells functionally resemble those in the lung. Contradictory data have been reported for the role of mast cells in fibrosis. A number of authors have proposed that mast cells are involved in fibrosis because they play a role in acute and chronic inflammation, which initiates it. In addition, mast cells are able to secrete histamine, heparin, and IL-4, which enhance the proliferation of fibroblasts. However, others [37,38], including us [39], have shown that mast cells play an antifibrotic role: for example, in animal models, such as mast cell-deficient Ws/Ws mice and rats. Okazaki et al. showed that induced fibrosis was more severe in mast cell-deficient rats than in wild-type rats [38].

Moreover, mast cells have been shown to be polarized in cancer, similarly to macrophages [40]. Anti-inflammatory mast cells express cytokines, such as IL-10, and their number is inversely associated with the severity of inflammation, whereas proinflammatory mast cells correspond to a proinflammatory setting. It is likely that anti-inflammatory mast cells express HLA-G, in particular, because (i) an association has been shown in several models between IL-10 levels and HLA-G expression and (ii) HLA-G has an anti-inflammatory action. HLA-G-expressing mast cells may be present at an early stage of the disease, during the inflammatory phase, to counteract inflammation, which is the first reaction to the lesion. In the literature, it was reported that Il-10, by reducing inflammatory response, may inhibit the proliferation and collagen synthesis of the myofibroblasts [41]. Indeed, IL-10 may play a protective role in alcoholic liver disease [42]. In contrast, higher serum levels of IL-10 were found in patients with IPF than normal subjects, and the highest level of IL-10 in the bronchoalveolar lavage was demonstrated in patients with IPF compared with sarcoidosis or hypersensitivity pneumonitis [43]. We could explain that by the less important inflammatory component. In renal fibrosis, it was demonstrated in a mouse model that a lack of IL-10 aggravated kidney inflammation and fibrosis [44]. In humans, treatment with local IL-10 immunotherapy associated with TGF-β antagonist improves chronic kidney disease [45].

Another relevant result is the observation of HLA-G^+^ cells in cell nodes, near the fibrotic regions in rare cases of alcoholic cirrhosis. These cells are morphologically characterized by a cluster of easily recognizable cells of small to medium size, suggesting a lymphoid aggregate. Their morphological characteristics are suggestive of follicles, which are structures formed mainly by B lymphocytes. Quadruple immunofluorescence in the nodes confirmed this hypothesis, as 68% of HLA-G^+^ cells co-expressed CD20, which is a specific marker of B lymphocytes (Table 3). Similar structures were also observed in IPF, with a similar result, showing 76% of HLA-G^+^ cells in nodes to be B lymphocytes (Table 6). Lymphoid neogenesis has been reported in fibrosis. Thus, under certain pathological conditions, such as persistent inflammation, the cellular aggregates may develop into a highly organized structure resembling secondary lymphoid tissue, i.e., tertiary lymphoid organs or ectopic lymphoid follicles [46]. Such lymphoid follicles contain T-cell-rich areas and distinct B-cell follicles with germinal centers [47]. The mechanism by which infiltrating B cells organize the ectopic follicle and germinal center is controlled by lymphotoxin-α_1_β_2_ and lymphoid chemokines, such as CC-chemokine ligand 19 (CCL19), CCL21, CXC-chemokine ligand 12 (CXCL12), and CXCL13, which regulate lymphocyte homing. In addition to lymphotoxin and chemokines, antigenic stimulation is also required to induce and maintain follicle formation. Such follicles were not observed in our cohort of hepatitis C virus-induced liver fibrosis and were only found in one of three cases of alcohol-induced liver fibrosis, suggesting a distinct stage of the disease. Indeed, the function of ectopic lymphoid organs and their correlation with inflammation and fibrosis is not yet clear. A number of studies have shown a novel and surprising role for B cells in regulating fibroblasts in fibrosis, in which their profibrotic effect is analogous to that of TGF-β and also enhanced by B-cell activating factor (BAFF) [48].

The relevance of the cellular source of HLA-G in fibrosis is not only descriptive but also functional. HLA-G has an inhibitory effect on the function of all types of lymphocytes [49] and dendritic cells [20,50] through its specific receptors, such as ILT2 and ILT4. The presence of HLA-G on these immune cells, in addition to being a marker of inflammation, is also a sign of an appropriate immune reaction by also decreasing inflammation. Indeed, HLA-G is known to play a protective role against exaggerated inflammatory reactions, as previously shown in septic shock [51]. In addition, we previously studied the reciprocal interaction between mast cells and hepatic stellate cells and showed that it leads to the attraction of mast cells and a significant decrease in collagen production by HSC cells through HLA-G production [39]. In addition, the expression of HLA-G by B cells in ectopic follicles may also contribute to counteract the profibrotic effect of B lymphocytes on myofibroblasts by inhibiting B cells via an autocrine mechanism.

## 4. Patients and Methods

### 4.1. Patients

A cohort of 41 liver transplant patients with alcohol-induced cirrhosis was studied. Patients were informed of the protocol, and the absence of opposition was obtained (Hospital Ethics Committee, notice No. 16.47).

Ten paraffin block samples of renal fibrosis and 10 of IPF (Hospital Ethics Committee, notice No. 16.123), completely and irreversibly anonymized, were studied in accordance with the principles of the Declaration of Helsinki.

The clinical and biological characteristics of the three cohorts are respectively summarized in Table 8, Table 9 and Table 10.

### 4.2. Methodology

#### Immunohistochemistry and Immunofluorescence

Tissues were derived from explanted liver and renal or pulmonary biopsy.

Paraffin-embedded serial sections (4 µm thick) were prepared, and standard histological staining, i.e., HES coloration and Sirius Red labeling of collagen, was performed. In parallel, immunohistochemistry and immunofluorescence were performed on serial sections of paraffin-embedded sections from the same block following deparaffinization and an antigen retrieval protocol.

Primary antibodies (mAbs) were as follows: monoclonal mouse anti-human HLA-G (Exbio, 4H84 2 µg/mL or 1:100, Vestec, Czech Republic), polyclonal rabbit anti-human CD117/c-kit, recognizing myeloid and mast cells (Dako, 1:200, Coppenhagen, Denmark), mouse monoclonal anti-human (hum) mast cell tryptase (clone AA1, Dako, 1:1000), specific for mast cells, monoclonal anti-human CD3 (Thermo Scientifics, SP7, 1:500, Waltham, MA, USA), specific for T lymphocytes, and monoclonal anti human CD20 (Dako, M0755, 1:600), specific for B lymphocytes.

Briefly, slides for immunohistochemistry were incubated with primary antibody in a Discovery Ultra (Roche, Meylan, France) automated system. Bound primary antibody was revealed using a biotinylated goat anti-mouse or anti-rabbit IgG secondary antibody (Vector, ABCYS, les Ulis, France, 1:700) and diamino-benzidine (DAB MAP detection kit, Roche, Meylan, France), followed by Mayer hematoxylin coloration.

To precisely determine the nature of the HLA-G^+^ cells, triple (DAPI, CD117, mast cell tryptase), quadruple (DAPI, HLA-G, CD117, mast cell tryptase), and quintuple (DAPI, HLA-G, CD3, CD20, mast cell tryptase) immunofluorescence staining were then performed on three representative cases of alcohol-induced cirrhosis and two representative cases of IPF. Revelation was performed using Discovery FAM, rhodamine, DCC, and Cy5 kits (Ventana Medical systems, Illkirch, France).

After staining, an image of the entire surface of the section was digitized at 20× or 40× magnification using a confocal scanner (Pannoramic Scanner, 3DHistech, Budapest, Hungary).

Immunohistochemistry and multiplex immunofluorescence staining were analyzed using HALO digital analysis software (V3.0.311). The software was trained to recognize fibrosis (Sirius Red) using the Area Quantification (V2.1.3) module, whereas the Fish-IF module (v1.2.2) was trained for the samples labeled with dyes. Whole sections of each sample were selected for analysis with the corresponding algorithm. Data were extracted to a spreadsheet software for analysis.

## 5. Conclusion

This work associated to our previous data experiments on the anti-protective role of the mast cells via HLA-G expression [39] suggest that mast cells play an antifibrotic and protective role via the expression of HLA-G in fibrotic situations. This role is reinforced by B lymphocytes expressing HLA-G in ectopic follicles. Overall, these findings suggest a protective role for HLA-G expressed by mast cells in fibrotic organs.

## Figures and Tables

**Table 1:** Enumeration of HLA-G^+^ and CD117^+^ cells/mm^2^ in the livers of patients with alcoholic cirrhosis (N = 41).

HLA-G/mm^2^		CD117/mm^2^	
Mean ± SD	Range	Mean ± SD	Range
555 ± 699	41–2686	200 ± 271	12–1113

**Table 2:** Nature of HLA-G^+^ cells expressed as a percentage following quadruple staining (DAPI/HLA-G/CD117/mast-cell tryptase) in alcoholic cirrhosis (N = 3).

	% of HLA-G^+^ Cells			
CD117- Mast Cell Tryptase-	CD117^+^	Mast Cell Tryptase^+^		
		Mast cell tryptase^+^ CD117^−^	Mast cell tryptase^+^ CD117^+^	Mast cell tryptase^+^ Total
49	0	13	38	51

**Table 3:** Nature of HLA-G^+^ cells according to the area of the liver in alcoholic cirrhosis (nodes and fibrosis).

% HLA-G Cells of Liver		
	Nodes	Fibrosis
CD3- CD20- mast cell tryptase	26	33
CD3+	3	0
CD20+	68	4
Mast cell tryptase +	3	63
CD31	0	0
CD1a	0	0

**Table 4:** Enumeration of HLAG^+^ cells, T and B lymphocytes, and mast cells in two different areas of the liver (nodes and fibrosis).

	Nodes	Fibrosis
HLA-G/mm^2^	849	99
CD3/mm^2^	368	57
CD20/mm^2^	3205	0
Mast-cell tryptase/mm^2^	77	62

**Table 5:** Enumeration of HLA-G^+^ cells and CD117 cells/mm^2^ in IPF.

Lung			
HLA-G/mm^2^		CD117/mm^2^	
Mean ± SD	range	Mean ± SD	range
135 ± 62	55–215	227 ± 134	79–521

**Table 6:** Nature of HLA-G cells expressed in percentage according to area of the lung (nodes and fibrosis).

	Nodes	Fibrosis
No CD3, no CD20, No Mast cell tryptase	17	34
CD3	0	3
CD20	76	0
Mast cell tryptase	7	63
CD31	0	0
CD1a	0	0

**Table 7:** Enumeration of HLA-G^+^ and CD117^+^ cells/mm^2^ in the kidney in renal fibrosis.

HLA-G/mm^2^		CD117/mm^2^	
Mean ± SD	range	Mean ± SD	range
133 ± 102	21–289	426 ± 207	90–773

**Table 8:** Clinical and biological characteristics of the cohort of alcoholic cirrhosis (N = 40). N is corresponding to the number of patients.

	Missing (N)	Mean (± std) or N (%)
**N = 42**		
**Age at transplant (yrs)**	0	60.1 ± 5.5
**Sex**	0	
Male		34 (80.9%)
Female		8 (19.1%)
**HCC**	0	17 (40.5%)
**Metavir**	1	
F0		1 (2.4%)
F1		2 (4.8%)
F2		0 (0%)
F3		0 (0%)
F4		38 (90.5%)
**Neuroinflammation**	19	
A0		16 (38.1%)
A1		7 (16.7%)
A2		0 (0%)
A3		0 (0%)
A4		0 (0%)
**MELD**	0	20.9 ± 6.9
**CHILD**	2	
A		4 (9.5%)
B		8 (19%)
C		28 (66.7%)
**CMV + Recipient**	0	16 (38.1%)
**CMV + Donor**	1	29 (69%)
**Rejection**	15	8 (19%)
**EAD**	0	12 (28.6%)

**Table 9:** Description of the cohort of IPF (N = 10). (N = 10). N corresponds to the number of patients.

	Missing (N)	Mean (± std) or N
Age at diagnosis(years)	0	65 ± 5
Sex		
Male (N)		9
Female (N)		1
Death (N)		3
Evolution time before death (year)	In live at time (N = 7)	4 ± 1
Comorbidities		
Body mass index	2	27.31 ± 2.8
Normal corpulence	5	
obesity	5	
Normal autoimmune tests	6	N = 3
Smoking status	3	7
Forming smoker		5
Current smoker		1
Never smoker		1
Vascular risk factors	6	4
Professional risk		1
Histology		
Common interstitial pneumonia equivalent to idiopathic pulmonary fibrosis N		10
Pulmonary function tests		
Tiffeneau’s ratio %	0	76 ± 28
FEV1 (forced expiratory volume in 1 s)%		84 ± 18
Vital capacity%	6	77 ± 5
Forced vital capacity (FVC)%	3	79 ± 24
Maximum expiratory flow rate%	4	97 ± 27
Total lung capacity %	0	67 ± 13
Residual volume %	4	61 ± 13
DLCO diffusion capacity of lung for carbon monoxide(N)	0	52 ± 17
Restrictive syndrome (N)		N = 9
Isolated capillary alveolo diffusion disorder (N)		N = 1
Restrictive syndrome and Capillary alveolo diffusion disorder (N)		N = 3

**Table 10:** Description of the cohort of renal fibrosis. N is corresponding to the number of patients.

	Missing (N)	Mean (± std) or N
Age at diagnosis (years)		63 ± 14
Sex		
Male (N)		4
Female (N)		6
Death (N)		0
Renal transplant(N)		2
**Comorbidities**		
Diabetes (N)		3
HTA (N)		2
Obesity (N)		3
LMMC (N)		1
Untreated rheumatoid arthritis (N)		
comorbid association (N)		3
**Symptoms at diagnosis**		
Chronic renal failure		8
progressive		2
severe		4
Acute renal failure		2
**Histology**		
Vascular nephropathy (N)		4
tubulo-interstitial nephropathy (N)		5
Immunoallergic nephropathy (N)		1
Sclerous glomerules and tubulointertitiel nephropathy (N)		1
Interstitial fibrosis and tubular atrophy (N)		
20%		3
30–40%		4
40–50%		1
>50%		1

## Data Availability

The data are stored on the H2P2 platform of the Biosit federative research structure.
